# Comparison of the effect of pressure loading on left ventricular size, systolic and diastolic function in canines with left ventricular dysfunction with preserved and reduced ejection fraction

**DOI:** 10.1186/1476-7120-6-57

**Published:** 2008-11-18

**Authors:** Steven J Lavine, Donald A Conetta

**Affiliations:** 1 Wayne State University, Detroit, MI 48202, USA; 2 University of Florida, Jacksonville, USA; 3 Cardiovascular Center, 655 West 8th Street, Jacksonville, FL 32209, USA

## Abstract

**Background:**

Decompensated heart failure may present with severe hypertension in patients with preserved (PreEF) or reduced left ventricular (LV) ejection fraction (RedEF) and is clinically indistinguishable. Previously, we demonstrated that arterial pressure elevation increases LV filling pressures in a canine model of chronic LV dysfunction with PreEF or RedEF. It is not clear whether any differences in hemodynamics, LV volume or performance, or diastolic function can be demonstrated between canines with PreEF or RedEF in response to arterial pressure elevation. We hypothesized that the LV systolic, diastolic, and hemodynamic response to pressure loading would be similar in RedEF or PreEF.

**Methods:**

We studied 25 dogs with chronic LV dysfunction due to coronary microsphere embolization with RedEF (35 ± 4%) and 20 dogs with PreEF (50 ± 3%). Arterial pressure was increased with methoxamine infusion and hemodynamics and echo-Doppler parameters of LV size, function, transaortic and transmitral pulsed Doppler prior to and with methoxamine infusion was obtained.

**Results:**

Though LV filling pressures were similar at baseline, LV size was larger (p < 0.01) and ejection fraction lower in dogs with RedEF (p < 0.001). With methoxamine, there were similar increases in LV size, LV pressures, and index of myocardial performance with the ejection fraction reduced similarly. Diastolic parameters demonstrated similar tau increases, E/A reduction, and diastolic filling shortening in RedEF and PreEF dogs. A similar extent of isovolumic contraction and relaxation times and index of myocardial performance prolongation occurred with pressure loading.

**Conclusion:**

Pressure loading in a canine model of LV dysfunction with PreEF and RedEF resulted in similar degrees of LV dilatation, increased filling pressures, and increased index of myocardial performance.

## Background

Decompensated heart failure is clinically indistinguishable in patients with either preserved or reduced left ventricular (LV) ejection fraction and may be accompanied by elevated arterial pressures. Although systolic dysfunction is often suspected, it is only after noninvasive imaging that the clinician discovers that the ejection fraction may be in the normal range. This may occur in up to 40–50% of patients depending on age and sex and is more common in elderly females with diabetes [1,2]. In addition, in patients with heart failure and LV systolic dysfunction, a significant though lower percentage of patients also have hypertension. Using a chronic canine model of LV dysfunction with either preserved or reduced ejection fraction induced by coronary microsphere embolization, we previously demonstrated that acute arterial pressure elevation results in marked elevation of LV filling pressures associated with prolonged relaxation and shortening of the diastolic filling period [3,4].

It is not entirely clear whether any differences in hemodynamics, LV volume or performance, or LV diastolic function can be demonstrated between canines with reduced or preserved LV ejection fraction in response to a stressor (e.g arterial pressure elevation). Although there have been some information in the literature regarding multiple levels of LV dysfunction, the model employed has been "tachypacing" induced heart failure, and the heart has been unloaded or contractile performance improved with dobutamine [5,6]. Little data has been generated using the coronary microsphere embolization model which produces a dilated, scarred left ventricle with reduced LV systolic function which has relevance to chronic coronary disease and LV dysfunction [7,8].

We hypothesized that the hemodynamic, LV volume and functional responses to the stress of pressure loading would be similar in a model of chronic LV dysfunction with reduced or preserved LV ejection fraction and would demonstrate similar elevations of LV filling pressures and volumes associated with similar direction and extent of abnormalities in indices describing diastolic function.

## Methods

The animals used in this study were maintained in accordance with the guidelines of the Committee on Animal Studies at Wayne State University School of Medicine and with the position of the American Heart Association on research animal use. The study was approved by the Wayne State University Animal Investigation Committee. Anesthesia was induced in 45 conditioned mongrel dogs (16–24 kg) with intramuscular morphine sulfate (1.5 mg/kg) and acepromazine (1.1 mg/kg) followed in 15 minutes by 30 mg/kg of intravenous ketamine hydrochloride. Maintenance anesthesia was produced by intravenous morphine sulfate (1.5 mg/kg/hr) and pentobarbital (3 mg/kg/hr). The dogs were intubated and artificially ventilated with a Harvard respirator using room air. Using fluoroscopic guidance, two 7 F high fidelity catheters (Millar Instruments) were introduced via the right carotid artery and advanced to the left ventricle and ascending aorta. A #8 multipurpose Judkins catheter was introduced through a sheath (Cordis) into the right femoral artery and advanced into the left coronary ostium. Continuous electrocardiographic monitoring was performed using lead II. At held end expiration, ECG, LV pressures, dP/dt, and central aortic pressures were obtained at 100 mm/s using an 8 channel physiologic recorder (Gould). Simultaneous 2 dimensional echocardiograms and Doppler were obtained from with the use of a phased array echocardiograph (Aloka). Transesophageal 4 and 2 chamber view with color flow were obtained from a 5 MHz biplane probe placed in the mid-esophagus with both transaortic and transmitral pulsed Doppler recordings were obtained from the LV outflow tract and from beyond the tips of the mitral leaflets in the left ventricle at 100 mm/s.

### Model of LV dysfunction with preserved LV ejection fraction

To test our hypothesis we employed a previously described canine model of LV dysfunction using coronary microsphere embolization [7,8]. In the development of this model, we discovered that the degree of LV dysfunction produced could be titrated based on microsphere number per injection, number of injections, and microsphere size. Re-embolization had been required to create models of moderate LV dysfunction when only minimal dysfunction had been previously created. Despite minimal systolic dysfunction, there had been remodeling with increased LV volumes, increased LV mass, and mild LV filling pressure elevation despite LV ejection fractions >50% [3,7,9].

LV dysfunction with preserved ejection fraction (PreEF) or reduced ejection fraction (RedEF) was induced by left main coronary artery plastic microsphere injections (50 and 80 micron) (3 M) injected in alternating boluses (1 cc) of 17,500 or 12,500 microspheres every 5–10 minutes until the peak positive dP/dt was reduced by >20% and the LV end diastolic pressure (LVEDP) was >10 mm Hg (for PreEF) or the peak positive dP/dt was reduced by >25% and LVEDP was >13 mm Hg (for RedEF)). Acute LV dysfunction (LV ejection fraction = 45–50% or 30–35%) was produced in 45–60 minutes with only minimal or mild mitral regurgitation (maximal jet area/left atrial area <20%). At 2 weeks post embolization, echocardiographic imaging was performed to assess LV systolic function. If the LV ejection fraction was >55% (for PreEF) or 40% (for RedEF), the animals were anaesthetized, intubated, and instrumented as above. Additional embolizations were performed reducing peak +dP/dT an additional 10–20% or until the LV ejection fraction was <50% (for PreEF) or <40% (for RedEF) following 45 minutes of stabilization. At this time, hemodynamics and Echo-Doppler imaging were repeated as above. This limited embolization approach ultimately leads to a chronic model of LV dysfunction and increased LVEDP characterized by patchy myocardial interstitial and replacement fibrosis (7–9) with an ejection fraction in the normal range (>50%) for PreEF or ejection fraction in the moderate dysfunction range (35–40%) for RedEF. Following 45 minutes of stable hemodynamics, the above parameters were repeated. The right carotid and femoral arteries were repaired and the dogs were allowed to recover without any dog succumbing in the PreEF group and 2 dogs in the RedEF group in the 1^st ^48 hours.

At 8 weeks post coronary microsphere embolization, the animals in both groups were anaesthetized, intubated, ventilated, instrumented, and imaged as above. Atrial pacing was instituted at least 5 beats above the baseline rate with a PR interval <160 msec. The above hemodynamics, cardiac outputs, echocardiographic imaging and Doppler recordings were obtained after 10 minutes of steady state pacing. Arterial pressure was increased with a methoxamine infusion to increase LV systolic pressure >40 mm Hg. Methoxamine was chosen as it increases arterial pressure without significant change in peak + dP/dT in this model. The above parameters were again obtained. The pacer was turned off and the dogs were permitted to return to their baseline hemodynamic state of chronic LV dysfunction.

### Hemodynamic, echocardiographic, and transmitral doppler measurements

For all stages and time periods, LV pressures, dP/dt, cardiac outputs, and aortic pressures were measured from the average of 3 consecutive cycles at held end expiration. Peak LV systolic pressure, LV minimal pressure, and LVEDP were measured. The time constant of LV pressure decline (tau) was calculated using the Weiss method (10). A frame-by-frame assessment of LV volumes using transesophageal apical views throughout the cardiac cycle was calculated using the biplane Simpson's rule from the average of 3 determinations. LV foreshortening rarely occurs in the canine as compared to humans. LV end diastolic volume was defined as the largest volume and end systolic volume as the smallest volume. LV ejection fraction was calculated as the difference between end diastolic volume and end systolic volume (stroke volume) divided by end diastolic volume. LV mass was calculated by the area length method. Effective arterial elastance was calculated as LV end systolic pressure/LV end systolic volume.

For all stages and time periods in both groups of dogs, all *Doppler indices *were measured from the average of 3 consecutive cycles at held end expiration. From transmitral Doppler indices, peak rapid filling velocity (E) and peak atrial filling velocity (A) were measured. The rapid filling deceleration time was calculated as the time interval from the peak rapid filling velocity to the time mitral flow decelerated to the zero baseline. The tracing was extrapolated to the zero baseline if atrial filling commenced prior to mitral flow fully decelerating to zero. The length of the diastolic filling period was obtained as the interval from beginning to the end of transmitral spectral tracing. When rapid and atrial filling velocity spectra demonstrated any degree of merging, the onset of atrial filling was defined at the point of the end of the p wave on the ECG. The time from the R wave to the end of the mitral time velocity spectrum and to the onset of the mitral time velocity spectrum (onset of flow) was obtained. The severity of mitral regurgitation was assessed as the ratio of maximal left atrial color flow jet area during systole divided by the simultaneous left atrial area.

### Index of myocardial performance

Index of myocardial performance (IMP) is defined as the sum of isovolumic contraction time and isovolumic relaxation time divided by the LV ejection time [3,4,11]. Using sequential pulse-wave Doppler tracing of the mitral inflow and transaortic outflow, IMP was calculated:

**IMP = (a-b)/b**

**a **= The period of time from the end of transmitral velocity spectrum of 1 beat to the onset of the transmitral velocity spectrum of the next beat. **b **= LVET is time interval from the onset of the pulsed Doppler transaortic spectrum to the end of the transaortic spectrum.

The isovolumic relaxation time (IRT) was measured as R wave to the onset of transmitral velocity spectrum minus the time from the R wave to the end of the aortic spectral tracing. The isovolumic contraction time (ICT) was measured as "a" minus the sum of LV ejection time and IRT.

LV pressure-volume composite plots were constructed for each stage and time period from mean LV pressures and LV volumes obtained throughout the cardiac cycle. An estimate of the operational LV chamber stiffness constant at end diastole was calculated using the approach of Marino, et al [12]. Essentially, the difference between LV pressure minimum and LVEDP was divided by the change in LV volume from the time of LV pressure minimum to end diastole.

All calculations were made off-line by the author (SL) and technical assistants (PP and VJ see acknowledgement) blinded to the dates of the studies, name of the dog, and experimental conditions. Intra-observer and inter-observer variability for LV volume was determined by selecting end diastolic and end systolic frames from the echocardiogram of 10 previously studied dogs. Each frame was analyzed 3 weeks apart by 2 observers (see acknowledgement). The average difference for end diastolic or end systolic volume was 2.2 cc's for intraobserver variability and 3.4 cc's for inter-observer variability.

### Statistics

All data was expressed as mean ± standard deviation. Differences between a variable among stages was assessed using analysis of variance for repeated measures. If the F statistic (p < 0.05) indicated a significant difference, then Tukey's test was utilized to determine where the significant differences existed. A p < 0.05 was considered significant.

## Results

Table 1 1summarizes parameters of LV size, systolic and diastolic function, and LV pressures at baseline and following induction of either chronic LV dysfunction with PreEF or RedEF in canines. LV size and mass increased, LV ejection fraction declined, LV filling pressures increased, and tau was prolonged in both groups following the induction of LV dysfunction. LV end diastolic and end systolic volumes were greater (p < 0.01) and the LV ejection fraction was further reduced in the RedEF group as compared to the PreEF group as per the design (p < 0.001). Despite, the lower ejection fraction and larger LV volumes, LVEDP and LV minimal pressures were similar in both the PreEF and RedEF groups. Transmitral Doppler parameters describing diastolic function revealed a reduction in deceleration time, and increases in IRT and ICT in both groups. IMP demonstrated an increase in both groups with a greater increase in the RedEF group (p < 0.05). This was due to a non-significantly greater IRT and ICT in the RedEF group. Mitral regurgitation was noted in 3 dogs with PreEF (minimal or mild in all 3 dogs; jet area/left atrial area = 4%, 5%, and 9%) and 5 dogs with RedEF (minimal or mild in 5 dogs; jet area/left atrial area = 2%, 5%, 7%, 8%, and 11%)

**Figure 1:**
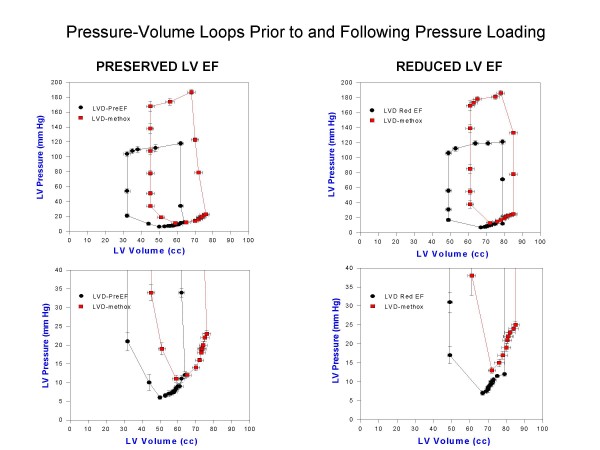
**Composite LV pressure-volume plots (mean ± standard error of the mean) at paced LV dysfunction and with peak methoxamine (LVD-methox) are shown for canines with LV dysfunction and preserved LV ejection fraction (LVD-PreEF) on the *left *and LV dysfunction with reduced ejection fraction (LVD Red EF) on the *right*.** Below are the expanded LV pressure-volume plots truncated at 40 mm Hg to demonstrate diastolic pressure differences more clearly. The pressure-volume curves are shifted rightward and upward for both groups of dogs. LV volumes are greater at both baseline LV dysfunction and with methoxamine in the group with reduced ejection fraction.

**Figure 2:**
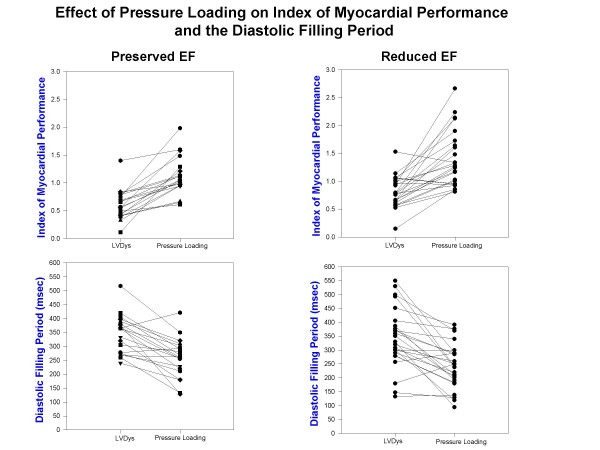
**Index of myocardial performance at baseline LV dysfunction (LVDys) and following pressure loading in dogs with LV dysfunction and preserved LV ejection fraction (left upper) and heart failure with reduced ejection fraction (right upper) demonstrates similar extent of increases with pressure loading.** Also, the diastolic filling period at baseline LV dysfunction and following pressure loading in dogs with LV dysfunction and preserved ejection fraction (left lower) and LV dysfunction with reduced ejection fraction (right lower) demonstrates a similar degree of shortening with pressure loading.

**Figure 3:**
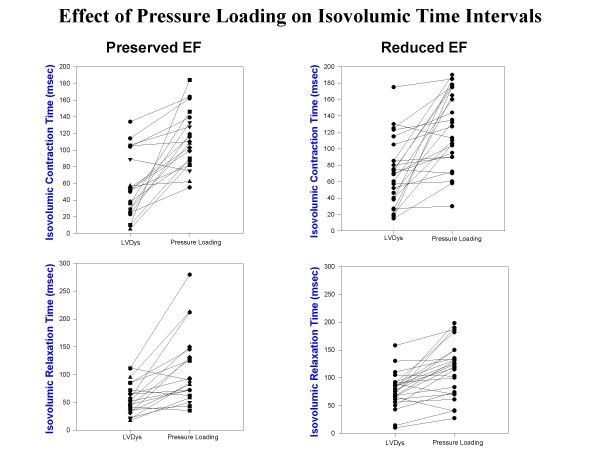
**Isovolumic contraction time at baseline LV dysfunction (LVDys) and following pressure loading in dogs with LV dysfunction and preserved ejection fraction (left upper) and LV dysfunction with reduced ejection fraction (right upper) demonstrates similar extent of increases with pressure loading.** Similarly, the isovolumic relaxation period at baseline LV dysfunction (LVDys) and following pressure loading in dogs with LV dysfunction and preserved ejection fraction (left lower) and LV dysfunction with reduced ejection fraction (right lower) demonstrates a similar degree of lengthening with pressure loading.

Table 2 1summarizes the results of arterial pressure incrementation with methoxamine in both groups. LV volumes increased with a reduction of LV ejection fraction in both groups. Stroke volume declined only in the RedEF group. LVEDP and LV minimal pressures, effective arterial elastance and chamber stiffness increased in both groups. Effective arterial elastance was lower in the RedEF group at baseline LV dysfunction (p < 0.05) and with pressure loading (p < 0.05) than in the PreEF group. Figure 1 displays composite LV pressure-volume plots (with mean ± standard error of the mean) prior to and following methoxamine infusions for PreEF (left) and RedEF (right) groups. Both groups demonstrate a similar rightward and upper shift of the pressure-volume curve from the baseline LV dysfunction plot. LV volumes (p < 0.01) were greater and LV ejection fraction (p < 0.001) were further reduced in the RedEF group than the PreEF group with arterial pressure elevation. Table 3 1summarizes the results of arterial pressure elevation in both groups with regard to diastolic filling parameters, IMP and its components. For both groups, E and E/A declined, the time to onset of mitral velocity was delayed, and was associated with shortening of diastolic filling, prolongation of IRT and ICT with marked increases in IMP. The IMP value with pressure loading was significantly more elevated in the RedEF group. Figure 2 and 3 summarizes the individual canine response in each group to pressure loading with regard to IMP and diastolic filling period (figure 2) and IRT and ICT (figure 3). The responses of each of these parameters to pressure loading were similar in both groups of dogs. Mitral regurgitation was noted in 5 dogs with PreEF with methoxamine infusion (mild in all; jet area/left atrial area = 6%, 6%, 8%, 10%, and 11%) and 8 dogs with ReEF (mild in all; 6%, 7%, 9%, 11%, 12%, 14%, 15% and 17%)

Table 4 1summarizes the changes in parameters with pressure loading in both the PreEF and RedEF groups. The direction, quantity, and percentage (data not shown) change in each parameter with arterial pressure elevation was similar in both canine LV dysfunction groups.

## Discussion

In this study we used a chronic canine model of LV dysfunction with elevated LVEDP's induced by coronary microsphere embolization with either a PreEF or RedEF to determine whether a stressor to LV performance would produce differences in hemodynamics, LV volume and systolic performance, and diastolic function. Methoxamine infusion was administered to increase arterial pressure >40 mm Hg above baseline. The effect of arterial pressure elevation on LV volumes, LV filling pressures, parameters of diastolic function, and IMP were determined.

This model of LV dysfunction is ideal as the level of LV dysfunction can be titrated based on the dosage of coronary microspheres and the number of different times the procedure is performed (7,8). Pressure loading resulted in identical increases in LV filling pressures associated with similar delays in the onset of diastolic filling, shortening of diastolic filling, and increases in isovolumic times. Consequently, LV filling volumes entered a poorly relaxed left ventricle at higher left atrial pressures at the time of mitral opening [3]. Changes in parameters of LV volume, systolic function, LV filling pressures and hemodynamics were similar as shown in table 4 despite LV volumes and IMP being larger and ejection fraction lower in the RedEF group prior to pressure loading. Changes in effective arterial elastance and operational LV chamber stiffness were similar as were qualitative changes in the composite LV pressure-volume plots. In summary, hemodynamic, LV volume, LV pressures, and diastolic changes were indistinguishable.

### Previous Literature

Using pressure-volume plots, the effect of pressure loading on LV dysfunction has been well described both clinically and experimentally [4,13]. However, only a modest amount of information is available for various levels of LV dysfunction and only in the model produced by rapid ventricular pacing [5,6]. Rahko [5] demonstrated that the position of the pressure-volume plot changed with varying levels of LV dysfunction as did the slope of the relation. However, he used inferior vena caval occlusion and the extent of LV dysfunction was not stable for an extended period of time as occurs with coronary microsphere embolization [7,8]. Pressure loading in the pacing model might have resulted in similar rightward shifts of the pressure-volume plot (with varying levels of LV dysfunction) with similar changes in effective chamber compliance as compared to the coronary microsphere model. The expected increases in LV volumes with both levels of LV dysfunction may induce pericardial constraint resulting in a similar rightward and upward shift in the pressure-volume plot [14]. Moe [6] studied the recovery from pacing induced LV dysfunction and demonstrated a downward shift in the velocity of circumferential shortening-end systolic stress plot with recovery. However, this study did not stress the left ventricle. Pressure-volume studies in pacing induced heart failure using afterload augmentation with phenylephrine and dobutamine augmentation also demonstrated a reduction in ventricular arterial coupling [15], but the effect of multiple levels of stable LV dysfunction were not addressed. Unfortunately, phenylephrine is also a positive inotrope initially unless beta blockade is used ([16] and personal observations). Using an isolated heart cross-perfused model of LV dysfunction previously produced by coronary microembolization, Todaka [17] demonstrated that the end diastolic pressure-volume relation was shifted rightward. However, multiple levels of LV dysfunction and afterload stress were not employed. Consequently, there is a paucity of studies examining the effects of afterload stress on LV systolic and diastolic performance with more than 1 level of LV dysfunction. This study served to help fill this void and points out that though the resting diastolic pressure-volume plots may differ in position in that more severe LV dysfunction is shifted rightward, the response to pressure loading is hemodynamically similar. The coronary microsphere model of LV dysfunction is uniquely suited to explore this issue as both varying levels of LV dysfunction can be produced for prolonged periods of time [7,8] and responses to pharmacologic interventions can be assessed [18]. Furthermore, this model can also be used to assess the force frequency relationship which may trend downward at higher rates with various causes of LV dysfunction [19].

Patients with decompensated heart failure commonly present in the emergency room with marked increases in arterial pressure. It is only after noninvasive evaluation of the LV ejection fraction that clinicians discover that the ejection fraction may be preserved or in the normal range. Heart failure with normal or PreEF has an increased proportion of elderly females and associated hypertension with rates of morbidity and mortality that have been described as either lower or equivalent to patients with RedEF [20-23]. It has been difficult to distinguish between PreEF and RedEF clinically in the presentation of acute decompensated heart failure. Acute decompensated heart failure may present as 1 of 3 presentations: cardiogenic shock, decompensation of chronic heart failure, and pulmonary edema with hypertension [24]. The ADHERE registry demonstrated at least 50% of patients and the Euro Heart Survey II described 34% of patients with decompensated heart failure presented with PreEF with lower mortality than patients with reduced ejection fraction though subsets of patients (reduced systolic blood pressure and renal insufficiency) had comparable mortality [22,25].

Studies attempting to differentiate PreEF from RedEF at the time of acute decompensation have been limited to surveys and registries. As the pathophysiology of this presentation is still not well understood, clinical or experimental trials addressing this issue have not yet been performed. As hypertension appears as an important co-morbidity, it stands to reason that it may participate in its pathogenesis. Reviews of therapy for decompensated heart failure have suggested normalizing blood pressure, diuresis, use of either angiotensin converting enzyme inhibitors or angiotensin receptor blockers, beta blockers, aldosterone inhibitors, and possibly nitrates for therapy [26-28]. Many of the above agents are useful in both heart failure with RedEF and PreEF especially agents that promote regression of LV hypertrophy, avoidance of tachycardia [29], reduction in interstitial collagen and reduction in the renin angiotensin system activity [30].

### Limitations

First, the appropriateness of the coronary microembolization model for the production of LV dysfunction is open to question. As the microsphere model of LV dysfunction results in diffuse fibrotic changes throughout the myocardium, its histopathology bears great similarity to the histopathology seen in hypertensive and diabetic cardiomyopathy [31,32], ischemic cardiomyopathy, and hypertensive heart disease with and without LV dysfunction. The applicability to all patients with heart failure and PreEF (ejection fraction >40%) is open to question. It has applicability to the group of patients with hypertensive heart disease as the etiology of heart failure. Alternatively, the use of a renovascular model of hypertension in aged dogs produces an excellent model (increased myocardial fibrosis) of LV dysfunction with PreEF for testing where hypertensive heart disease is the etiology of heart failure [33]. This study does not address the issue of the appropriateness of whether the etiology and pathogenesis of heart failure with PreEF or RedEF should be different. This study is simply assessing the hemodynamic and LV volume and function response to pressure loading in experimental groups based on LV ejection fraction. Certainly, the etiology may be the same or different for both heart failure with PreEF and RedEF. However, there is similarity of hemodynamic, LV volume, systolic and diastolic responses with PreEF vs RedEF.

Second, one must raise the question whether pressure loading is the appropriate stressor. Earlier studies using volume loading increased LV size and LV filling pressures but lengthened LV ejection time [34]. Patients with LV dysfunction often have increased IMP's due to lengthening of the isovolumic indices and shortening of LV ejection time [11,35]. Also, pressure loading increases IMP by lengthening the isovolumic indices and insignificantly shortening LV ejection time [3,4], a finding that mirrors the expected findings in a patient with severe LV systolic function. Finally, a substantial number of patients present with pulmonary edema and hypertension who rapidly respond to lowering of their arterial pressure and have PreEF or RedEF [20,22]. However, methoxamine may not be a pure afterload stressor as 1 study has suggested that it may increase preload to a greater extent than angiotensin II [36]. In addition, an alpha adrenergic agonist may also increase contractility [37] though peak positive dP/dT was unchanged in this study with methoxamine infusion.

Third, as this is an experimental study, the applicability of these findings and relation to humans is always a limitation. The general anesthesia utilized may influence the results, and the results may differ with conscious canines. However, this anesthesia regimen has been used in numerous studies in our laboratory and results in lower arterial pressures and LV filling pressures, which may lessen the impact of our intervention. Nevertheless, the experimental data cited here clearly indicates that there is little hemodynamic difference to pressure loading based on LV ejection fraction. This is clearly paralleled clinically by our experience in the emergency room when patients who are severely hypertensive and present with acute decompensated heart failure. There are few clinical cues as to whether the LV ejection fraction is normal or reduced. The importance of blood pressure control may assume even greater importance in patients with hypertensive heart disease and symptoms of heart failure.

## Conclusion

Pressure loading in a canine model of LV dysfunction with PreEF or RedEF resulted in similar degrees of LV dilatation, increased filling pressures, and increased IMP associated with similar delays in isovolumic relaxation and contraction despite a larger left ventricle and a lower ejection fraction in the RedEF group.

## Abbreviations

LV: left ventricular; PreEF: preserved ejection fraction; LVEDP: left ventricular end diastolic pressure; RedEF: reduced ejection fraction; E: peak rapid filling velocity; A: peak atrial filling velocity; IMP: index of myocardial performance; IRT: isovolumic relaxation time; ICT: isovolumic contraction time.

## Competing interests

The authors declare that they have no competing interests.

## Authors' contributions

SL designed the study, carried out the experimental protocol, analyzed the data, and wrote the manuscript. DC assisted in the drafting and revising of the manuscript, provided criticism of the results as they pertain to existing literature, and approved the final version submitted.

## Supplementary Material

Additional file 1 Click here for file
