# Supplementary material for: Comparison of the effect of pressure loading on left ventricular size, systolic and diastolic function in canines with left ventricular dysfunction with preserved and reduced ejection fraction
Source: Cardiovasc Ultrasound. 2008 Nov 18;6:57. doi: 10.1186/1476-7120-6-57 (PMC2626582; doi:10.1186/1476-7120-6-57)
Supplement: Additional file 1 [file 1476-7120-6-57-S1.doc]

Table 1: Parameters of LV Size, Systolic and Diastolic Function, and LV Pressures in Canines with LV Dysfunction with Preserved and Reduced Ejection Fractions

PRESERVED LV EF REDUCED EF

|  | **Baseline** | **LVDys** | **Baseline** | **LVDys** |
| --- | --- | --- | --- | --- |
| EDV (cc) | 51+13 | 65+11** | 53+11 | 76+9*** |
| ESV (cc) | 19+9 | 32+9*** | 21+8 | 49+6*** |
| Stroke Volume (cc) | 33+11 | 32+10 | 33+9 | 26+8** |
| EF (%) | 61+7 | 50+3*** | 60+9 | 35+4*** |
| LV Mass (g) | 71+16 | 86+15* | 72+14 | 91+21** |
| LV Minimal Pressure (mm Hg) | 2+2 | 6+3** | 2+2 | 7+3*** |
| LVEDP (mm Hg) | 5+3 | 12+5*** | 5.8+2.1 | 12+4*** |
| LV Systolic Pressure (mm Hg) | 117+15 | 118+14 | 118+11 | 121+20 |
| Tau (msec) | 23+3 | 39+11*** | 22+3 | 37+8*** |
| E/A | 1.8+0.4 | 1.5+0.7 | 1.7+0.4 | 1.5+0.7 |
| DCT (msec) | 217+27 | 137+51*** | 211+22 | 146+45*** |
| ICT (msec) | 36+21 | 65+35** | 35+22 | 74+26*** |
| IRT (msec) | 41+19 | 57+29** | 40+21 | 75+31*** |
| DFP (msec) | 371+149 | 344+69 | 366+158 | 343+108 |
| RR (msec) | 696+67 | 677+71 | 683+78 | 673+94 |
| IMP | 0.38+0.13 | 0.65+35** | 0.36+0.12 | 0.81+0.27*** |

**Abbreviations**: LVDys=LV dysfunction; EDV=end diastolic volume; ESV=end systolic volume; EF=ejection fraction; LVEDP=LV end diastolic pressure; DCT=deceleration time; ICT=isovolumic contraction time; IRT=isovolumic relaxation time; DFP=diastolic filling period; RR=cycle length; IMP=index of myocardial performance.

**Statistics**: *p<0.05, **p<0.01, ***p<0.001 vs Baseline

Table 2: Parameters of LV Size, Systolic Function, LV Pressures, and Arterial and LV Stiffness in Canines with LV Dysfunction with Preserved and Reduced Ejection Fractions Prior to and Following Pressure Loading

**PRESERVED LV EF REDUCED EF**

|  | **LVDys** | **LVDys-PL** | **LVDys** | **LVDys-PL** |
| --- | --- | --- | --- | --- |
| **EDV (cc)** | 64+13 | 76+10** | 77+8 | 85+8** |
| **ESV (cc)** | 32+10 | 45+11*** | 49+9 | 61+12*** |
| **Stroke Volume (cc)** | 31+10 | 30+9 | 29+8 | 23+8* |
| **EF (%)** | 50+3 | 41+5*** | 35+4 | 28+6*** |
| **Peak +dP/dT (mm Hg/s)** | 1736+303 | 1789+341 | 1609+206 | 1630+201 |
| **LV Minimal Pressure (mm Hg)** | 6+2 | 11+4*** | 7+3 | 13+3*** |
| **LVEDP (mm Hg)** | 12+5 | 23+7*** | 12+4 | 25+6*** |
| **LV Systolic Pressure (mm Hg)** | 118+14 | 187+23*** | 121+20 | 186+30*** |
| Tau (msec) | 38+11 | 50+13** | 37+8 | 51+12*** |
| Effective Arterial Elastance (mmHg/cc) | 3.1+0.6 | 4.1+0.8** | 2.2+0.8 | 3.1+1.1** |
| **LV Chamber Stiffness (mmHg/cc)** | 0.50+0.28 | 1.11+0.60** | 0.61+0.31 | 1.04+0.51** |
| **RR Interval (msec)** | 634+87 | 634+87 | 638+102 | 638+102 |

**Abbreviations:** LVDys=LV dysfunction with pressure loading; see table 1

**Statistics:** **p<0.01, ***p<0.001 vs LVDys

Table 3: Parameters of Diastolic Function and Index of Myocardial Performance in Canines with LV Dysfunction with Preserved and Reduced Ejection Fractions Prior to and Following Pressure Loading

**PRESERVED LV EF REDUCED EF**

|  | **LVDys** | **LVDys-PL** | **LVDys** | **LVDys-PL** |
| --- | --- | --- | --- | --- |
| **E (cm/s)** | 82+22 | 63+8** | 86+25 | 66+21** |
| **E/A** | 1.5+0.6 | 0.9+0.7** | 1.5+0.8 | 1.0+0.6** |
| **DCT (msec)** | 139+48 | 114+49* | 149+40 | 118+51* |
| **RMVO (msec)** | 329+40 | 402+65** | 354+40 | 413+59** |
| **DFP (msec)** | 348+62 | 258+71*** | 349+98 | 242+80*** |
| **IMP** | 0.64+0.33 | 1.10+0.34*** | 0.82+0.29 | 1.49+0.5*** |
| **IRT (msec)** | 59+33 | 110+64*** | 74+37 | 114+55** |
| **ICT (msec)** | 63+37 | 98+46** | 75+29 | 120+49** |
| **LVET (msec)** | 190+20 | 190+14 | 187+28 | 175+25 |

**Abbreviations:** RMVO=time interval from R wave to onset of mitral flow; see Table 1 for other abbreviations

**Statistics**: *p<0.05, **p<0.01, ***p<0.001 vs LVDys

**Table 4: Alterations in LV Size, Function, Filling Pressure, Diastolic**

**Filling Parameters, and IMP with Pressure Loading**

|  | **Preserved EF** | Reduced EF |
| --- | --- | --- |
| **EDV (cc)** | +11+12 | +9+11 |
| **ESV (cc)** | +13+13 | +12+11 |
| **Stroke Volume (cc)** | -2+9 | -6+12 |
| **EF (%)** | -9+5 | -7+4 |
| **LV Minimal Pressure (mm Hg)** | +5+3 | +6+2 |
| **LVEDP (mm Hg)** | +11+8 | +13+7 |
| **Tau (msec)** | +12+6 | +14+5 |
| **Effective Arterial Elastance mmHg/cc)** | +1.0+0.9 | +0.9+1.0 |
| **LV Chamber Stiffness (mmHg/cc)** | +0.61+0.60 | +0.53+0.48 |
| **E (cm/s)** | -19+23 | -20+24 |
| **E/A** | -0.61+0.54 | -0.49+0.36 |
| **DCT (msec)** | -25+26 | -32+33 |
| **RMVO (msec)** | +73+58 | +59+52 |
| **DFP (msec)** | -86+54 | -101+65 |
| **IMP** | +0.45+0.38 | +0.65+0.37 |
| **IRT (msec)** | +53+47 | +39+36 |
| **ICT (msec)** | +33+41 | +46+44 |

Abbreviations: See table 1,2, and 3

Statistics: No significant differences between Reduced and Preserved EF
